# Efficacy and safety of idebenone tablets in patients with post-stroke cognitive impairment: a real-world study

**DOI:** 10.3389/fneur.2025.1599501

**Published:** 2025-10-20

**Authors:** Yanqi Shao, Xinyu Zhao, Zunchun Xie, You Xie, Shunyuan Guo

**Affiliations:** ^1^Department of Neurology, Zhejiang Provincial People’s Hospital, Hangzhou, China; ^2^Department of Neurology, The First Affiliated Hospital of Zhengzhou University, Zhengzhou, China; ^3^Department of Neurology, The First Affiliated Hospital of Nanchang University, Nanchang, China; ^4^Department of Neurology, Xiangxi Tujia and Miao Autonomous Prefecture People’s Hospital, Jishou, China

**Keywords:** idebenone, post-stroke cognitive impairment, real-world study, cognitive function, RWS

## Abstract

**Objective:**

To evaluate the efficacy, safety, and adherence of idebenone tablets in improving cognitive function among patients with post-stroke cognitive impairment in a real-world setting.

**Methods:**

This single-arm, multicenter, real-world observational study enrolled 3,755 patients with post-stroke cognitive impairment from 342 hospitals across China between January 2020 and December 2024. Patients received idebenone (30 mg three times daily) for 3 months. Cognitive function was assessed using MoCA and MMSE at baseline and months 1, 2, and 3. Treatment effectiveness was categorized as markedly effective (≥80% improvement), effective (30–79% improvement), or ineffective (<30% improvement).

**Results:**

Of the 3,755 patients (mean age 60.7 ± 10.4 years; 58.5% male), 61.8% had hypertension and 48.4% had ischemic stroke. The total effectiveness rate increased progressively from 10.9–13.0% at month 1 to 37.4–38.2% at month 3 for both MoCA and MMSE scores (*p* < 0.001). MoCA scores improved from 14.6 ± 5.1 at baseline to 17.6 ± 6.2 at month 3, while MMSE scores increased from 14.2 ± 4.6 to 17.5 ± 6.2. High medication adherence (≥80%) was achieved by 96.9% of patients. Only mild adverse events were reported in less than 2% of patients, with no severe adverse events documented.

**Conclusion:**

This real-world study suggests that three-month idebenone therapy provides meaningful improvements in cognitive function among patients with post-stroke cognitive impairment, with excellent safety and adherence profiles. However, the observational, single-arm design without a control group means that observed improvements cannot be definitively attributed to the intervention alone. Further randomized controlled trials are warranted to confirm these findings and optimize treatment protocols.

## Introduction

Stroke, defined by the World Health Organization (WHO) as a clinical syndrome of rapidly developing focal (or global) disturbance of cerebral function lasting more than 24 h or leading to death, is one of the leading causes of mortality and morbidity worldwide (1). Within China, stroke remains the top cause of death, placing an immense burden on healthcare systems (2, 3). Such figures highlight the urgency of improving prevention, treatment, and rehabilitation strategies for stroke, particularly ischemic stroke, which accounts for approximately 80% of all stroke cases (4).

Despite advances in acute stroke management, a substantial proportion of patients surviving an ischemic stroke experience residual neurocognitive dysfunctions that can impair quality of life (5). One of the most common and challenging sequelae is post-stroke cognitive impairment (PSCI). PSCI can be defined as any degree of cognitive deficit occurring within 6 months after a stroke, encompassing a spectrum from mild cognitive impairment to dementia-like syndromes (6). Recent estimates reveal that up to 80.97% of stroke survivors may develop varying degrees of cognitive impairment (7). These deficits, often involving memory, attention, executive function, and visuospatial abilities, hamper patients’ capacity to resume independent daily activities, reduce their motivation to engage in rehabilitation, and affect emotional well-being (8). Cognitive deficits are frequently accompanied by motor and sensory impairments, generating a complex clinical picture that challenges rehabilitation efforts. Traditional rehabilitation strategies for post-stroke deficits include physical therapy, occupational therapy, balance training, speech therapy, and psychological interventions (7). However, the effectiveness of these interventions may be limited by reduced patient compliance due to fatigue, depression, and other comorbidities, especially when the rehabilitation process is prolonged. Given the multifactorial nature of cognitive recovery, pharmacological treatments aimed at enhancing brain metabolism, improving cerebral blood flow, or modulating neurotransmission have been explored as an adjunct to conventional rehabilitation (6).

Idebenone (chemical name: 2,3-dimethoxy-5-methyl-6-(10-hydroxydecyl)-1,4-benzoquinone) is a synthetic analog of coenzyme Q10. Unlike coenzyme Q10, idebenone has a shorter, modified side chain containing an additional hydroxyl group, which enhances its bioavailability and facilitates its passage across biological membranes, including the blood–brain barrier (9). Early pharmacologic studies have shown that idebenone improves mitochondrial electron transport and boosts ATP production, helping to stabilize or restore neuronal function under oxidative stress (9). Studies in various neurological conditions indicate that idebenone can promote neuronal energy metabolism, mitigate neuronal damage from reactive oxygen species, and potentially foster recovery of cognitive and motor functions (9, 10). Some clinical data suggest that idebenone may be beneficial for cognitive impairment associated with a variety of conditions, including Alzheimer’s disease (AD), mitochondrial encephalomyopathies, and post-ischemic brain injury (9, 11, 12). Li et al. (10) observed that among patients with movement disorders, idebenone treatment led to significant symptomatic relief with few adverse reactions, while another group (Zou et al.) demonstrated that idebenone administration was associated with improvements in both cognitive and behavioral symptoms in psychiatric disorders (11). These positive outcomes are thought to stem from idebenone’s capacity to enhance mitochondrial function, thereby increasing glucose utilization in the brain and promoting neuronal recovery (9).

In the context of post-stroke cognitive impairment, the rationale for using idebenone lies in its antioxidative properties and its role in boosting cellular energy metabolism. Neurons compromised by ischemia may suffer from inadequate ATP supply and excess oxidative free radicals, both of which hamper synaptic activity and cognitive processing. By improving mitochondrial respiration and reducing lipid peroxidation, idebenone may help attenuate neuronal damage, stabilize neural membrane function, and improve neurotransmitter release (9, 13). Enhanced neuronal survival and connectivity, in turn, could facilitate better cognitive recovery.

However, despite these promising mechanisms of action, real-world data on the efficacy and safety of idebenone specifically in post-stroke cognitive impairment are still limited. Many of the previously published findings come from controlled clinical trials with relatively narrow inclusion criteria, which may not reflect the complexities of routine practice. In real-world clinical settings, patients often have multiple comorbidities such as hypertension, hyperlipidemia, diabetes, or cardiac disorders, and they vary widely in age, functional status, and socioeconomic circumstances (2, 14). Real-world evidence (RWE) studies, which examine treatments in naturalistic medical environments, can therefore complement clinical trial findings by identifying potential variations in treatment response, adherence patterns, and adverse events among more diverse patient populations (15).

Real-world studies may also illuminate how treatment adherence—defined as the degree to which patients follow prescribed treatment regimens—affects clinical outcomes. For pharmacotherapeutic agents that require long-term administration, such as idebenone in cognitive impairment, understanding how non-adherence impacts efficacy and safety outcomes is crucial (16). In the context of PSCI, consistent use of cognitive-enhancing medications may be particularly important during the window of neuroplasticity following stroke, when rehabilitation and pharmacological interventions can have the most pronounced effects on long-term outcomes (17).

Furthermore, investigating the timing of administration—how soon after a stroke idebenone therapy is initiated and for how long it is continued—may yield critical insights into optimizing treatment protocols. Some studies on neuroprotective agents in stroke have suggested that earlier administration can lead to better outcomes, but more evidence is required specifically for idebenone (18). Therefore, an extensive, multicenter, real-world observational study can provide practical guidance for clinicians by shedding light on how idebenone is used in actual clinical practice. This approach can confirm or refute prior controlled-trial results, highlight patient subgroups that may benefit most, and reveal any unanticipated side effects (19). Such data can also inform public health strategies on resource allocation, by clarifying the cost-effectiveness of idebenone therapy in stroke rehabilitation (20).

This study aims to investigate the efficacy, safety, and adherence of idebenone tablets in improving cognitive function among patients with post-stroke cognitive impairment. Through a comprehensive evaluation of large-scale, multicenter data, we seek to enrich the current evidence base and support more targeted therapeutic strategies in routine stroke care.

## Methods

### Study design

This study was conducted as a single-arm, multicenter, real-world observational investigation aimed at evaluating both the effectiveness and safety of idebenone tablets in patients diagnosed with post-stroke cognitive impairment. The study period encompassed January 1, 2020, to December 31, 2024. Because real-world data research is intended to capture the natural course of clinical management, no additional interventions or procedures, apart from routine care, were imposed on the participants.

Patients meeting the eligibility criteria were followed over 3 months of idebenone therapy. During this period, routine clinical assessments were documented, including cognitive evaluations, adverse event reporting, and medication adherence data. By collecting information from multiple centers located in different regions, the study aimed to reflect the diversity of clinical settings and patient populations.

### Study population

The study population comprised patients with clinically confirmed post-stroke cognitive impairment who used idebenone tablets as part of their routine treatment between January 1, 2020, and December 31, 2024. Stroke was defined in accordance with the diagnostic standards laid out in the *Chinese Guidelines for Clinical Management of Cerebrovascular Diseases (2019)* (3). Cognitive impairment was identified based on either patient or caregiver reporting of cognitive difficulties within 3 months after the stroke event, or by the judgment of an experienced clinician. Participants were included if they were at least 18 years of age, had no severe mental disorders or established dementia, and had sufficiently complete medical records to allow for outcome assessment.

In line with the requirement to avoid bullet points except for inclusion/exclusion criteria, the following are the eligibility parameters:

#### Inclusion criteria

(1) Patients meeting the diagnostic criteria for stroke as stipulated in the *Chinese Guidelines for Diagnosis and Treatment of Acute Ischemic Stroke 2018*.
(2) Evidence of cognitive decline within 3 months of stroke onset, either by patient/caregiver report or clinical judgment.
(3) Age ≥18 years, no restriction on sex.
(4) Receipt of idebenone tablets for the treatment of post-stroke cognitive impairment within the specified study period (January 1, 2020, to December 31, 2024).

#### Exclusion criteria

(1) Incomplete clinical data that would preclude outcome evaluation.
(2) Severe psychiatric disorders or known dementia prior to stroke onset.

### Outcome measures

The study’s primary outcome measure was the change in cognitive function from baseline to the third month of treatment, as assessed by two instruments: the Montreal Cognitive Assessment (MoCA) and the Mini-Mental State Examination (MMSE). These scales were chosen for their widespread clinical use and validated reliability in detecting cognitive impairment.

Treatment effectiveness was categorized based on changes in MoCA and MMSE scores between baseline and the third month of therapy. If both MoCA and MMSE improved by at least 80% of the possible improvement from baseline, the outcome was classified as “markedly effective.” If the improvement reached at least 30% but below 80%, the outcome was termed “effective.” When improvements did not reach 30%, the outcome was recorded as “ineffective.” The overall effectiveness rate was defined as the sum of “markedly effective” plus “effective” cases, divided by the total number of evaluable patients (21).

Secondary outcome measures included repeated cognitive evaluations at 1 month and 2 months post-baseline, rates and characteristics of adverse events, dosage and duration of idebenone therapy, and overall medication adherence. Adherence was operationalized as the ratio of actual days of idebenone usage to the total expected days (3 months). Patients with adherence of 80% or greater were considered adherent.

Adverse events were documented in medical records as part of routine care and classified according to type (e.g., gastrointestinal, neurological), frequency, and severity. Any unexpected or serious adverse events were closely monitored and reported in accordance with institutional regulations.

### Data collection procedures

Data were obtained retrospectively from electronic health records or paper charts across multiple sites. Investigators at each center performed thorough chart reviews using a standardized electronic Case Report Form (eCRF). Data points included demographic details (age, sex, education), relevant comorbidities (hypertension, hyperlipidemia, diabetes, heart disease), stroke subtype (hemorrhagic vs. ischemic vs. transient ischemic attack), time since stroke onset, baseline cognitive scores, and subsequent cognitive assessments at one, two, and 3 months. All cognitive assessments were conducted face-to-face by trained clinical staff at each participating center. In cases of clearly inconsistent results, clinicians were advised to re-assess to minimize inter-rater variability in this study.

Medication records were reviewed to determine idebenone dosage, frequency, and duration of use. In most participating centers, the standard regimen for idebenone was 30 mg per dose, three times per day. Any deviation from this regimen was recorded for further analysis.

To minimize bias, original patient identifiers were removed and replaced by a study-specific code, ensuring patient privacy. Data validation checks were applied to detect inconsistencies or missing information, and queries were resolved by returning to the original records. When a discrepancy could not be resolved, the data point was treated as missing.

### Statistical analysis

After data extraction, all analyses were carried out using SAS 9.4 statistical software (SAS Institute, Cary, NC, United States). Continuous variables such as age, MoCA and MMSE scores were summarized as mean ± standard deviation or as median with interquartile range (25–75%), depending on their distribution. Categorical variables were summarized as frequencies and percentages. To evaluate changes in MoCA and MMSE scores from baseline to follow-up, paired-sample t-tests or Wilcoxon signed-rank tests (for non-normally distributed data) were conducted. Changes in the proportion of patients deemed “effective” or “markedly effective” were assessed via McNemar’s test, which is specifically tailored for paired categorical data. Patients were divided into two groups based on adherence (≥80% vs. <80%). Between-group differences in demographic, clinical, and outcome variables were assessed by independent-samples t-tests for continuous data and chi-square tests for categorical data. Adverse events were tabulated by type and severity. Incidence rates were calculated and, if sample sizes allowed, logistic regression was performed to explore associations between demographic factors (e.g., age, comorbidities) and the likelihood of experiencing an adverse event. A two-sided *p*-value less than 0.05 was considered statistically significant for all analyses. Missing values were handled by pairwise deletion.

### Data management and quality assurance

Throughout the study, data management followed national regulations on patient confidentiality and data integrity. Each participating center was responsible for collecting, de-identifying, and entering data into the eCRF. Medical staff members performed an initial check to confirm consistency with the patient’s source documents, and a statistician performed subsequent validations. Discrepancies were resolved by referring back to the original medical records; if still unresolved, relevant data fields were left as missing. Audit trails were maintained to document any changes made to the dataset. The principal investigator at the coordinating center oversaw periodic quality control checks, ensuring that at least 10% of patient records from each site were randomly audited for accuracy. This effort was intended to mitigate selection bias and ensure robust data collection.

### Ethical considerations

Because this project was an observational study that used data collected during routine medical care, the requirement for written informed consent was either waived or modified, depending on local institutional review board (IRB) policies. Where mandated, patients or their legal representatives provided informed consent for the use of their data for research purposes. The study protocols adhered to the Declaration of Helsinki and national guidelines on the ethical conduct of research involving human participants. No additional interventions outside routine care were imposed, and no changes to medication regimens were made specifically for study purposes.

## Results

### Patient demographic characteristics and distribution

Table 1 summarizes the baseline characteristics of the included patients. Among the 3,755 participants, 2,198 were male (58.5%) and 1,557 were female (41.5%). Mean age was 60.7 ± 10.4 years (range, 24–95), and the median age was 61.0. Notably, 16.6% of the patients had completed high school, and 11.0% had attained education levels at or above college/technical school. Regarding comorbidities, 61.8% had hypertension, 17.9% had hyperlipidemia, 13.8% had diabetes, and 5.5% had heart disease.

**Table 1:** Baseline characteristics of the study population.

Variable	Overall (*N* = 3,755)	Adherence ≥80% (*N* = 3,639)	Adherence <80% (*N* = 116)	*p*-value
Sex (*n*, %)				0.08
Male	2,198 (58.5%)	2,121 (58.3%)	77 (66.4%)	
Female	1,557 (41.5%)	1,518 (41.7%)	39 (33.6%)	
Age				0.02
Mean ± SD	60.7 ± 10.4	60.8 ± 10.5	58.7 ± 10.1	
Range	24.0–95.0	24.0–95.0	35.0–91.0	
Median (Q1–Q3)	61.0 (53.0–68.0)	61.0 (53.0–68.0)	58.5 (51.0–65.0)	
Education level (*n*, %)				<0.001
Illiterate	553 (14.7%)	551 (15.1%)	2 (1.7%)	
Primary school	1,323 (35.2%)	1,312 (36.1%)	11 (9.5%)	
Junior high school	844 (22.5%)	817 (22.5%)	27 (23.3%)	
High school	623 (16.6%)	590 (16.2%)	33 (28.4%)	
College/Technical or Above	412 (11.0%)	369 (10.1%)	43 (37.1%)	
Medical history (*n*, %)				
Hypertension	2,319 (61.8%)	2,254 (61.9%)	65 (56.0%)	0.20
Hyperlipidemia	673 (17.9%)	656 (18.0%)	17 (14.7%)	0.35
Diabetes	520 (13.8%)	487 (13.4%)	33 (28.4%)	<0.001
Heart disease	208 (5.5%)	207 (5.7%)	1 (0.9%)	0.03
Stroke subtype (*n*, %)				<0.001
Hemorrhagic stroke	863 (23.0%)	853 (23.4%)	10 (8.7%)	
Transient ischemic attack	1,075 (28.6%)	973 (26.7%)	102 (87.9%)	
Ischemic stroke	1817 (48.4%)	1813 (49.9%)	4 (3.4%)	
Duration of stroke (*n*, %)				<0.001
1 year	1,685 (44.9%)	1,601 (44.0%)	84 (72.4%)	
1–5 years	1847 (49.2%)	1817 (49.9%)	30 (25.9%)	
5–10 years	197 (5.2%)	195 (5.4%)	2 (1.7%)	
>10 years	26 (0.7%)	26 (0.7%)	0 (0.0%)	
Baseline MoCA Score				<0.001
Mean ± SD	14.6 ± 5.1	15.0 ± 4.6	2.4 ± 2.1	
Range	1.0–25.0	1.0–25.0	1.0–19.0	
Median (Q1–Q3)	15.0 (12.0–18.0)	15.0 (12.0–18.0)	2.0 (2.0–2.0)	
Baseline MMSE Score				<0.001
Mean ± SD	14.2 ± 4.6	14.5 ± 4.1	2.5 ± 1.2	
Range	1.0–24.0	1.0–24.0	1.0–10.0	
Median (Q1–Q3)	15.0 (11.0–17.0)	15.0 (12.0–17.0)	2.0 (2.0–3.0)	
Region (*n*, %)				<0.001
Northeast	24 (0.6%)	24 (0.7%)	0 (0.0%)	
North China	37 (1.0%)	37 (1.0%)	0 (0.0%)	
East China	1,409 (37.5%)	1,294 (35.6%)	115 (99.1%)	
South China	210 (5.6%)	210 (5.7%)	0 (0.0%)	
Central China	1983 (52.8%)	1982 (54.5%)	1 (0.9%)	
Northwest	92 (2.5%)	92 (2.5%)	0 (0.0%)	

In terms of stroke diagnosis, hemorrhagic stroke accounted for 23.0% of patients, transient ischemic attacks for 28.6%, and ischemic stroke for 48.4%. Roughly half of the participants (49.2%) had a stroke duration (from initial diagnosis) of 1–5 years, while 44.9% had experienced stroke for less than 1 year.

At baseline, the average MoCA score was 14.6 ± 5.1, and the average MMSE score was 14.2 ± 4.6. Disaggregating the results by medication adherence (≥80% vs. <80%) yielded statistically significant differences in several variables, including age, education level, history of diabetes, history of heart disease, stroke subtype, disease duration, and baseline MoCA/MMSE scores (*p* < 0.05).

### Medication use information

All patients received idebenone at a dose of 30 mg per administration, three times daily. The study tracked the total days of idebenone use for each 1-month period over a total of 3 months (Table 2). For all three time intervals (treatment months 1, 2, and 3), the most common duration of use was 30 days, accounting for approximately 73–74% of patients each month.

**Table 2:** Idebenone medication use characteristics.

Variable	Statistic (*N* = 3,755)
Treatment Month 1	
Number of days of idebenone use	
10	113 (3.0%)
12	2 (0.1%)
25	5 (0.1%)
26	1 (0.0%)
28	122 (3.2%)
29	4 (0.1%)
30	2,774 (73.9%)
31	9 (0.2%)
32	707 (18.9%)
35	18 (0.5%)
Treatment month 2	
Number of days of idebenone use	
10	115 (3.1%)
25	14 (0.4%)
28	128 (3.4%)
30	2,763 (73.6%)
31	6 (0.2%)
32	712 (19.0%)
33	4 (0.1%)
35	13 (0.2%)
Treatment Month 3	
Number of days of idebenone use	
10	116 (3.1%)
25	19 (0.5%)
27	2 (0.1%)
28	107 (2.8%)
30	2,783 (74.1%)
31	7 (0.2%)
32	716 (19.1%)
35	5 (0.1%)
Overall adherence rate (%)	
<80	116 (3.1%)
≥ 80	3,639 (96.9%)

A total of 3,639 patients (96.9%) had a medication adherence rate of 80% or higher, whereas 116 patients (3.1%) demonstrated an adherence rate below 80%.

### Efficacy assessments

**Figure 1:**
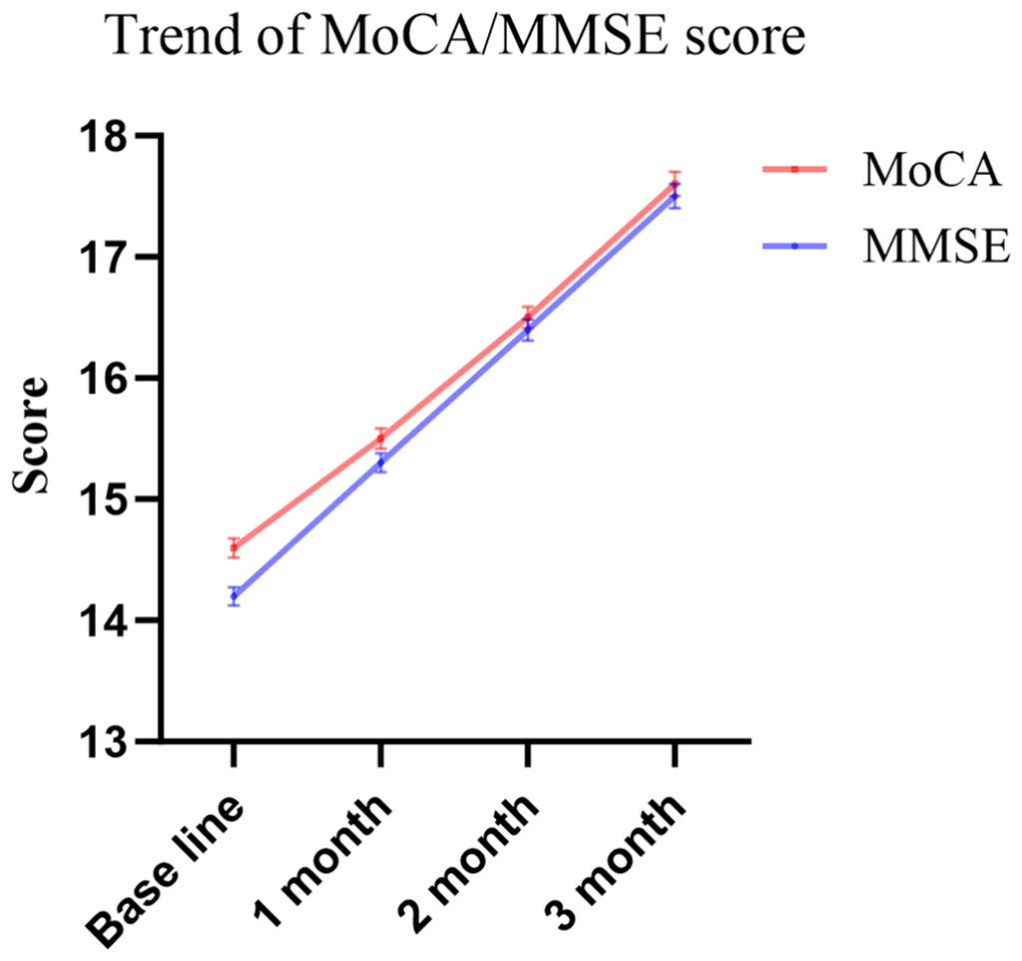
Trend of MoCA/MMSE score (Mean±SE).

Cognitive outcomes were evaluated at one, two, and 3 months post-baseline. A progressive improvement in MMSE and MoCA total scores was observed throughout the treatment period. Mean scores exhibited a steady upward trend, rising from between 14 and 15 at baseline to between 17 and 18 upon completion of 3 months of therapy(Figure 1). Table 3 presents both the absolute scores in MoCA and MMSE at each time point, as well as the rates of “markedly effective” and “effective” responses.

**Table 3:** Distribution of cognitive outcomes at each follow-up.

Variable	Statistic (*N* = 3,755)
Month 1	
MoCA (mean ± SD)	15.5 ± 5.3 (range 2–29)
MMSE (mean ± SD)	15.3 ± 5.0 (range 2–29)
MoCA effectiveness	
Ineffective	3,345 (89.1%)
Markedly effective	87 (2.3%)
Effective	323 (8.6%)
Total effective	410 (10.9%)
MMSE effectiveness	
Ineffective	3,267 (87.0%)
Markedly effective	71 (1.9%)
Effective	417 (11.1%)
Total effective	488 (13.0%)
Month 2	
MoCA (mean ± SD)	16.5 ± 5.6 (range 2–30)
MMSE (mean ± SD)	16.4 ± 5.6 (range 2–30)
MoCA effectiveness	
Ineffective	2,764 (73.6%)
Markedly effective	151 (4.0%)
Effective	840 (22.4%)
Total effective	991 (26.4%)
MMSE effectiveness	
Ineffective	2,691 (71.7%)
Markedly effective	155 (4.1%)
Effective	909 (24.2%)
Total effective	1,064 (28.3%)
Month 3	
MoCA (mean ± SD)	17.6 ± 6.2 (range 1–30)
MMSE (mean ± SD)	17.5 ± 6.2 (range 1–30)
MoCA effectiveness	
Ineffective	2,352 (62.6%)
Markedly effective	297 (7.9%)
Effective	1,106 (29.5%)
Total effective	1,403 (37.4%)
MMSE effectiveness	
Ineffective	2,321 (61.8%)
Markedly effective	303 (8.1%)
Effective	1,131 (30.1%)
Total effective	1,434 (38.2%)

Comparisons using chi-square tests revealed a statistically significant increase in the percentage of patients classified as “effective” or “markedly effective” at 3 months compared to 1 month (*p* < 0.001) and 2 months (*p* < 0.001; Table 4).

**Table 4:** Comparisons of cognitive outcomes over time.

Variable	Month 3 vs. Month 1	Month 3 vs. Month 2
MoCA	Total Effective at Month 3: 1403 (37.4%)	Total Effective at Month 3: 1403 (37.4%)
	Total Effective at Month 1: 410 (10.9%)	Total Effective at Month 2: 991 (26.4%)
Chi-Square (*χ*^2^)	717.0	104.1
*p*-value	<0.001	<0.001
MMSE	Total Effective at Month 3: 1434 (38.2%)	Total Effective at Month 3: 1434 (38.2%)
	Total Effective at Month 1: 488 (13.0%)	Total Effective at Month 2: 1064 (28.3%)
Chi-Square (*χ*^2^)	625.8	82.1
*p*-value	<0.001	<0.001

### Adverse events and safety

Adverse event data indicated that idebenone was well-tolerated overall. Gastrointestinal complaints (e.g., nausea, mild abdominal discomfort) were the most commonly reported, but these occurred in fewer than 2% of patients. Transient headaches or dizziness were also documented in a small subset, generally resolving spontaneously. No severe adverse events definitively attributable to idebenone were reported.

### Summary of key findings

In this large real-world analysis of 3,755 patients with post-stroke cognitive impairment, approximately 97% achieved an adherence rate of 80% or higher over 3 months. Cognitive outcomes showed progressive improvement from Month 1 to Month 3 as reflected in MoCA and MMSE scores, with total effectiveness rates increasing from around 10–13% at Month 1 to about 37–38% at Month 3. The low prevalence of adverse events corroborates the overall safety and tolerability of idebenone in this population.

## Discussion

Stroke remains one of the most serious health challenges globally, with high morbidity, mortality, and long-term disability rates, especially in lower- and middle-income countries (16). Post-stroke cognitive impairment complicates recovery in a substantial proportion of survivors, posing enormous burdens not only for patients and their families but also for healthcare systems (17).

A gradual increase in the total effectiveness rate from 10.9–13.0% at Month 1 to approximately 37–38% at Month 3, as measured by MoCA and MMSE, suggests that prolonged administration of idebenone can yield clinically meaningful improvements. This observation aligns with the understanding that neural plasticity continues for weeks to months post-stroke (18). Early improvements in cognitive scales at the one-month mark might reflect partial symptomatic relief or residual effects of acute-phase treatments, whereas sustained improvements over 3 months are more indicative of neurorecovery processes aided by enhanced mitochondrial function (9).

Several potential mechanisms may underlie these improvements. Idebenone exerts antioxidant effects and augments ATP production by facilitating electron flow in the mitochondrial respiratory chain (9). Ischemic stroke leads to mitochondrial dysfunction, reduced oxidative phosphorylation, and overproduction of free radicals, all contributing to neuronal damage (19). By partially restoring normal mitochondrial activity, idebenone might curb ongoing injury and support synaptic plasticity, particularly in peri-infarct regions (20). Additionally, idebenone’s ability to traverse the blood–brain barrier distinguishes it from other coenzyme Q10 analogs that may have limited central nervous system penetration (9).

Furthermore, improvements in MoCA and MMSE indicate potentially broad effects on multiple cognitive domains, including attention, executive function, and memory (22). This broad-spectrum enhancement is consistent with the possibility that idebenone positively influences fundamental neuronal processes rather than merely modulating a single neurotransmitter pathway (23). However, it is crucial to recognize that the absolute proportion of patients achieving “markedly effective” status by Month 3, while higher than at earlier time points, is still not a majority. This outcome highlights the multifactorial nature of PSCI and the need for integrated approaches that combine pharmacotherapy with rehabilitation, psychosocial support, and management of vascular risk factors (24).

In the present study, the overall similarity between baseline MMSE and MoCA scores appears somewhat divergent from the cognitive profile typically observed in pure vascular cognitive impairment (VCI) (25). Classically, VCI is characterized by prominent deficits in executive and visuospatial functions, with relative preservation of memory and calculation, often leading to lower MoCA than MMSE scores. However, the cognitive phenotype of PSCI encompasses a wider spectrum than pure VCI, which may help explain our findings. Two factors may be relevant: first, approximately 80% of all dementia cases are characterized by mixed pathology, encompassing both AD and vascular pathologies in older stroke populations (26). The possible explanation for this phenomenon is that a considerable proportion of elderly stroke patients who appear clinically normal may already have significant underlying AD pathology in the brain prior to the stroke event. Following the occurrence of stroke, these latent pathological processes become activated or unmasked, leading to a cognitive phenotype that more closely resembles the AD pattern—such as prominent memory impairment (27). Co-existing AD-type pathologies, may modulate cognitive profiles and attenuate the dissociations between MoCA and MMSE often seen in purely vascular cases. Second, the large sample size (*n* = 3,755) and multi-center design included different cognitive subtypes: the MoCA was more sensitive for detecting non-amnestic deficits, whereas the MMSE showed higher specificity for amnestic deficits (28). Therefore, the baseline similarity between the two screening instruments in our cohort may be seen as reflecting the true clinical and pathological heterogeneity of PSCI in a large, unselected population, underscoring the complexity of cognitive profiles in real-world settings.

The majority of patients (96.9%) maintained high adherence (≥80%), which is a positive finding, as medication adherence is frequently problematic in long-term treatment regimens. Several factors may have contributed to the high adherence rate. First, many patients were recruited from larger stroke centers or hospitals that might have had robust education programs and follow-up mechanisms (29). Second, idebenone’s convenient oral administration schedule (e.g., 30 mg three times per day) and its generally favorable side effect profile may have improved tolerability and acceptance (30). Third, the real-world nature of the study may have selected for patients who were more motivated or whose caregivers actively participated in the treatment process.

Nonetheless, the study also identified a small subgroup (3.1%) with adherence below 80%. These patients exhibited different baseline characteristics, such as higher prevalence of diabetes, younger average age, or distinct stroke subtypes. Lower adherence might arise from cost considerations, difficulties in obtaining prescription refills, or negative perceptions of the medication’s effectiveness (31). Exploring these barriers in depth could help tailor patient education or policy changes to ensure consistent medication usage, thereby maximizing benefits (32).

Controlled clinical trials and smaller studies have explored idebenone in a variety of neurological contexts, often reporting beneficial effects on cognition or neuropsychiatric symptoms (9–11). Our results corroborate these earlier findings in a specific PSCI population but contribute novel real-world evidence that might be more generalizable to routine clinical practice. Real-world data are often messier and include patients with multiple comorbidities, polypharmacy, and other complicating factors. Hence, the relatively robust efficacy signal and low rate of adverse events in this large sample offer reassurance about idebenone’s practical utility (15).

The current findings also resonate with research on other mitochondrial enhancers or antioxidants tested in stroke populations, such as citicoline or acetyl-L-carnitine, which have shown modest to moderate cognitive benefits (33). However, direct comparisons among different agents remain challenging because of variations in study design, endpoints, and patient populations. Moreover, the broader category of neuroprotective drugs has historically faced difficulty demonstrating clear functional benefits in stroke clinical trials, potentially due to timing of administration and methodological constraints (34). Our data suggest that a three-month course might be more beneficial than shorter regimens, underscoring the relevance of drug exposure duration.

The progressive improvement in cognitive scores over 3 months implies that idebenone may require sustained administration to yield maximal neurorestorative effects. Stroke pathophysiology involves acute infarction or hemorrhage, followed by a subacute phase of spontaneous partial recovery, and eventually a chronic stage during which plasticity remains but is often reduced (21). Pharmacologic interventions that support metabolic recovery and synaptic reorganization could be particularly vital in the subacute phase, typically spanning the first few months after the initial insult (16). Real-world observational data in this study highlight that incremental benefits can emerge cumulatively, supporting the recommendation for a minimum treatment duration of at least 3 months in appropriate candidates.

Earlier interventions might still be advantageous. Some patients may have begun idebenone therapy within days or weeks post-stroke, while others might have delayed onset due to logistical or clinical reasons. Although our study did not systematically analyze the effect of the precise start time of idebenone in relation to stroke onset, future research could examine whether an early start confers additional benefit in neuroplastic windows (17).

Safety is a critical determinant of whether a therapy can be broadly implemented in older populations who often have multiple comorbidities. Our study identified only a small proportion of mild to moderate adverse events, mainly gastrointestinal or transient neurological complaints. No serious adverse events definitively linked to idebenone were reported. This aligns with previous safety data indicating that idebenone is typically well-tolerated (9, 12).

Nonetheless, real-world pharmacovigilance remains essential, as rare but serious events could manifest in larger cohorts. Clinicians must also remain cautious about potential drug–drug interactions, particularly with medications frequently prescribed in post-stroke patients (e.g., antihypertensives, antiplatelets, anticoagulants).

Despite the strengths of this study’s large sample size and multicenter design, several limitations must be acknowledged. First, as a single-arm observational study, there was no control group receiving a placebo or alternative treatment, which restricts the capacity to attribute improvements exclusively to idebenone. Some or all of the gains in MoCA and MMSE could be partly due to spontaneous recovery or concurrent rehabilitation therapies (20). Comparing results to historical controls or using matching techniques could partially mitigate this concern, but randomized controlled trials remain the gold standard for causality inferences (23). Second, neuroplasticity after stroke is dynamic, and the benefits of metabolic support may vary with the timing of therapy initiation. Due to the retrospective nature of the data collection, we could not systematically analyze the impact of the timing of therapy initiation relative to the stroke event. This is a critical unanswered question, as prior evidence suggests that interventions introduced during the subacute phase of heightened neuroplasticity may confer greater benefits (17, 18), while later treatment can still yield meaningful improvements as cognitive impairment remains highly prevalent long after stroke (7). Our study nevertheless provides value by reflecting real-world practice, in which treatment is often initiated at variable time points, and demonstrates that idebenone is safe and associated with cognitive improvements across this diverse spectrum. Future prospective studies should stratify outcomes by timing of initiation to identify the optimal therapeutic window for PSCI management. Third, data on concomitant medications and non-pharmacological interventions were not systematically collected. Patients may have been receiving other treatments, such as other cognitive enhancers, antidepressants, or intensive rehabilitation programs, which could have influenced cognitive scores. Fourth, We identified potential selection biases, as the population was drawn from hospitals that had the resources and motivation to systematically document patients receiving idebenone. Patients with minimal access to healthcare or those who did not tolerate the medication early on might have been underrepresented. Fifth, adherence calculations were based on prescription refills and self-reported use. While this method is common in real-world research, it does not guarantee ingestion of the medication as prescribed. Memory deficits inherent to PSCI could further complicate accurate self-reporting. Finally, we used two cognitive assessments (MoCA and MMSE) that are well-validated but may not capture specific cognitive deficits, such as executive function, in sufficient detail. Moreover, due to the lack of subject stratification, the mean scores of the MoCA and MMSE were closely aligned in the overall population. Using specialized tools like the Trail Making Test or the Stroop Test might provide additional information in future studies.

## Conclusion

This real-world, multicenter, single-arm observational study highlights a notable trend of cognitive improvement in patients with post-stroke cognitive impairment who received idebenone over a three-month period. The progressive increase in effectiveness from the first to the third month underscores the potential of idebenone to support neural recovery, possibly through enhanced mitochondrial function and antioxidant effects. High adherence rates demonstrate that the medication is generally well-accepted by patients, while the low incidence of adverse events points to a favorable safety profile. Nonetheless, the absence of a comparator group and certain methodological constraints limit definitive conclusions. Future controlled trials, combined with mechanistic investigations, may further substantiate the role of idebenone in PSCI management. For clinicians, however, these findings offer valuable real-world guidance, suggesting that a three-month course of idebenone might be a viable adjunct to conventional stroke rehabilitation, especially for patients who can maintain strong medication adherence and have no contraindications. By aligning pharmacological and rehabilitative interventions, healthcare professionals can strive to improve the functional and cognitive outcomes of the growing population of stroke survivors worldwide.

## Data Availability

The raw data supporting the conclusions of this article will be made available by the authors, without undue reservation.

## References

[1] Sacco RL Kasner SE Broderick JP. An updated definition of stroke for the 21st century: a statement for healthcare professionals from the American Heart Association/American Stroke Association. Stroke. (2013) 44:2064–89. doi: 10.1161/STR.0b013e318296aeca, PMID: 23652265 PMC11078537

[2] Wang W Jiang B Sun H Ru X Sun D Wang L. Prevalence, incidence, and mortality of stroke in China: results from a nationwide population-based survey of 480,687 adults. Circulation. (2017) 135:759–71. doi: 10.1161/CIRCULATIONAHA.116.025250, PMID: 28052979

[3] Chinese Stroke Association. Chinese guidelines for clinical management of cerebrovascular diseases. Beijing, China: People's Medical Publishing House (2019).

[4] Yitshak Sade M Novack V Ifergane G Horev A Kloog I. Air pollution and ischemic stroke among young adults. Stroke. (2015) 46:3348–53. doi: 10.1161/STROKEAHA.115.010992, PMID: 26534971

[5] Feigin VL Krishnamurthi RV Parmar P. Update on the global burden of ischemic and hemorrhagic stroke in 1990-2013: the GBD 2013 study. Neuroepidemiology. (2015) 45:161–76. doi: 10.1159/000441085, PMID: 26505981 PMC4633282

[6] Ren QG Liang B Meng XS. Overview of clinical diagnostic criteria for vascular cognitive impairment. J Pract Med. (2019) 35:1–3. doi: 10.3969/j.issn.1006-5725.2019.01.001

[7] Jokinen H Melkas S Ylikoski R. Post-stroke cognitive impairment is common even after successful clinical recovery. Eur J Neurol. (2015) 22:1288–94. doi: 10.1111/ene.12743, PMID: 26040251

[8] Zhang JS. Discussion on humanized management in psychiatric nursing administration. China Urban Rural Enterprise Hyg. (2018) 33:102–4. doi: 10.16286/j.1003-5052.2018.11.040

[9] Yang XY Han BF Cui SZ Akinobu N. Pharmacological effects and clinical application of idebenone in the treatment of ischemic cerebrovascular diseases. New Drugs Clin Rem. (1993) 1:11–4.

[10] Li HY Zeng XQ Li J Huang LL Lan XY. Observation of the therapeutic effect of idebenone in Parkinson’s disease and related nursing research. Contemp Nurse. (2020) 27:26–7. doi: 10.19792/j.cnki.1006-6411.2020.17.011

[11] Zou C Wang Y Liu LM Ou SH Zhang F Liu JJ. Efficacy and mechanism of idebenone on cognitive dysfunction in patients with schizophrenia. Med Forum. (2022) 26:19–21. doi: 10.19435/j.1672-1721.2022.29.007

[12] Wang H Wang X Wang W Feng D. Effects of idebenone on cognitive function and serum biomarkers in patients with amnestic mild cognitive impairment. Eur J Med Res. (2024) 29:600. doi: 10.1186/s40001-024-02184-w, PMID: 39696692 PMC11657116

[13] Zhang X Li J. Clinical efficacy of idebenone in stroke patients with mild cognitive impairment, and its effect on regional homogeneity of resting-state functional magnetic resonance imaging of the brain. Trop J Pharm Res. (2022) 21:1557–64. doi: 10.4314/tjpr.v21i7.28

[14] Prust ML Forman R Ovbiagele B. Addressing disparities in the global epidemiology of stroke. Nat Rev Neurol. (2024) 20:207–21. doi: 10.1038/s41582-023-00921-z, PMID: 38228908

[15] Jaksa A Wu J Jónsson P. Organized structure of real-world evidence best practices: moving from fragmented recommendations to comprehensive guidance. J Comp Eff Res. (2021) 10:711–31. doi: 10.2217/cer-2020-0228, PMID: 33928789

[16] Feigin VL Brainin M Norrving B Martins S Sacco RL Hacke W. World stroke organization (WSO): global stroke fact sheet 2022. Int J Stroke. (2022) 17:18–29. doi: 10.1177/17474930211065917, PMID: 34986727

[17] Salbach NM Mountain A Lindsay MP Blacquiere D McGuff R Foley N. Canadian stroke best practice recommendations: virtual stroke rehabilitation interim consensus statement 2022. Am J Phys Med Rehabil. (2022) 101:1076–82. doi: 10.1097/PHM.0000000000002062, PMID: 35767008

[18] Wang C. The role of neuromodulation to drive neural plasticity in stroke recovery: a narrative review. Brain Netw Modul. (2022) 1:2–8. doi: 10.4103/2773-2398.339171

[19] Tuo Q Zhang S Lei P. Mechanisms of neuronal cell death in ischemic stroke and their therapeutic implications. Med Res Rev. (2022) 42:259–305. doi: 10.1002/med.21817, PMID: 33957000

[20] Mousaei Ghasroldasht M Seok J Park HS Liakath Ali FB al-Hendy A. Stem cell therapy: from idea to clinical practice. Int J Mol Sci. (2022) 23:2850. doi: 10.3390/ijms23052850, PMID: 35269990 PMC8911494

[21] Fu QY Wang B Xu Y. Clinical efficacy of idebenone in treating cognitive impairment in post-stroke patients. China Med Guide. (2021) 19:107–9. doi: 10.15912/j.cnki.gocm.2021.26.040

[22] Quinn TJ Richard E Teuschl Y. European stroke organisation and European academy of neurology joint guidelines on post-stroke cognitive impairment. Eur Stroke J. (2021) 6:3883–920. doi: 10.1177/23969873211042192, PMID: 34476868

[23] Craig L Hoo ZL Yan TZ Wardlaw J Quinn TJ. Prevalence of dementia in ischaemic or mixed stroke populations: systematic review and meta-analysis. J Neurol Neurosurg Psychiatry. (2022) 93:180–7. doi: 10.1136/jnnp-2020-325796, PMID: 34782389 PMC8784999

[24] D’Souza CE Greenway MRF Graff-Radford J Meschia JF. Cognitive impairment in patients with stroke. Semin Neurol. (2021) 41:75–84. doi: 10.1055/s-0040-1722217, PMID: 33418591

[25] Cumming TB Churilov L Linden T Bernhardt J. Montreal cognitive assessment and Mini-mental state examination are both valid cognitive tools in stroke. Acta Neurol Scand. (2013) 128:122–9. doi: 10.1111/ane.12084, PMID: 23425001

[26] Brayne C Richardson K Matthews FE Fleming J Hunter S Xuereb JH. Neuropathological correlates of dementia in over-80-year-old brain donors from the population-based Cambridge city over-75s cohort (CC75C) study. J Alzheimer's Dis. (2009) 18:645–58. doi: 10.3233/JAD-2009-1182, PMID: 19661624

[27] Jansen WJ Ossenkoppele R Knol DL Tijms BM Scheltens P Verhey FR. Prevalence of cerebral amyloid pathology in persons without dementia: a meta-analysis. JAMA. (2015) 313:1924–38. doi: 10.1001/jama.2015.4668, PMID: 25988462 PMC4486209

[28] Chen X Han Y Zhou J Ma M Liu X. Diagnostic accuracy of cognitive screening tools under different neuropsychological definitions for poststroke cognitive impairment. Brain Behav. (2020) 10:e01671. doi: 10.1002/brb3.1671, PMID: 32621406 PMC7428509

[29] GBD 2019 Stroke Collaborators. Global, regional, and national burden of stroke and its risk factors, 1990-2019: a systematic analysis for the global burden of disease study 2019. Lancet Neurol. (2021) 20:795–820. doi: 10.1016/S1474-4422(21)00252-0, PMID: 34487721 PMC8443449

[30] Fens T Zhou G Postma MJ van Puijenbroek EP van Boven JFM. Economic evaluations of chronic obstructive pulmonary disease pharmacotherapy: how well are the real-world issues of medication adherence, comorbidities and adverse drug-reactions addressed? Expert Opin Pharmacother. (2021) 22:923–35. doi: 10.1080/14656566.2021.1873953, PMID: 33435700

[31] Katsanos AH Hart RG. New horizons in pharmacologic therapy for secondary stroke prevention. JAMA Neurol. (2020) 77:1308–17. doi: 10.1001/jamaneurol.2020.2494, PMID: 32716473

[32] Bansilal S Castellano JM Garrido E Wei HG Freeman A Spettell C. Assessing the impact of medication adherence on long-term cardiovascular outcomes. J Am Coll Cardiol. (2016) 68:789–801. doi: 10.1016/j.jacc.2016.06.005, PMID: 27539170

[33] Alvarez-Sabín J Román GC. Citicoline in vascular cognitive impairment and vascular dementia after stroke. Stroke. (2011) 42:S40–3. doi: 10.1161/STROKEAHA.110.606509, PMID: 21164117

[34] Gladstone DJ Black SE Hakim AM. Toward wisdom from failure: lessons from neuroprotective stroke trials and new therapeutic directions. Stroke. (2002) 33:2123–36. doi: 10.1161/01.str.0000025518.34157.51, PMID: 12154275

